# Cellular and Functional Physiopathology of Bull Sperm With Altered Sperm Freezability

**DOI:** 10.3389/fvets.2020.581137

**Published:** 2020-10-23

**Authors:** Mustafa Hitit, Muhammet Rasit Ugur, Thu Tran Nhat Dinh, Dishnu Sajeev, Abdullah Kaya, Einko Topper, Wei Tan, Erdogan Memili

**Affiliations:** ^1^Department of Animal and Dairy Sciences, Mississippi State University, Starkville, MS, United States; ^2^Department of Animal Genetics, Kastamonu University, Kastamonu, Turkey; ^3^Department of Reproduction and Artificial Insemination, Selcuk University, Konya, Turkey; ^4^URUS Group LP, Madison, WI, United States; ^5^Department of Basic Sciences, College of Veterinary Medicine, Mississippi State University, Starkville, MS, United States

**Keywords:** sperm biology, bull, freezability, cellular, functional parameters

## Abstract

The objective of this study was to ascertain the cellular and functional parameters as well as ROS related changes in sperm from bulls with varied sperm freezability phenotypes. Using principal component analysis (PCA), the variables were reduced to two principal components, of which PC1 explained 48% of the variance, and PC2 explained 24% of the variance, and clustered animals into two distinct groups of good freezability (GF) and poor freezability (PF). In ROS associated pathophysiology, there were more dead superoxide anion positive (Dead SO+) sperm in GF bulls than those in PF (15.72 and 12.00%; *P* = 0.024), and that Dead SO+ and live hydrogen positive cells (live H_2_O_2_+) were positively correlated with freezability, respectively (*R*^2^ = 0.55, *P* < 0.0130) and (r_s_ = 0.63, *P* = 0.0498). Related to sperm functional integrity, sperm from PF bulls had greater dead intact acrosome (DIAC) than those from GF bulls (26.29 and 16.10%; *P* = 0.028) whereas sperm from GF bulls tended to have greater live intact acrosome (LIAC) than those from PF bulls (64.47 and 50.05%; *P* = 0.084). Sperm with dead reacted acrosome (DRAC) in PF bulls were greater compared to those in GF (19.27 and 11.48%; *P* = 0.007). While DIAC (*R*^2^ = 0.56, *P* = 0.0124) and DRAC (*R*^2^ = 0.57, *P* < 0.0111) were negatively correlated with freezability phenotype, LIAC (*R*^2^ = 0.36, *P* = 0.0628) was positively correlated. Protamine deficiency (PRM) was similar between sperm from GF and PF bulls (7.20 and 0.64%; *P* = 0.206) and (r_s_ = 0.70, *P* = 0.0251) was correlated with freezability. Sperm characteristics associated with cryotolerance are important for advancing both fundamental andrology and assisted reproductive technologies across mammals.

## Introduction

Bull fertility, regarded as the ability of viable sperm to fertilize the egg and then support early embryonic development, is essential for the propagation of species, and it is an important economic trait for animal breeding programs. There is a global need for modern and sustainable food farming of animals such as cattle for the production of quality milk and meat to feed the world. The use of fresh or frozen sperm through artificial insemination (AI) combined with genomic selection has accelerated the genetic improvement of livestock (1, 2). Cryopreservation, defined as the successive stabilization of temperature with the process of dehydration, freezing, thawing, is a useful and profitable procedure for long-term storage of sperm in domestic animals and humans (3). Cryopreservability or freezability of sperm is crucial for the success of AI, which has become the preferred method of breeding in the cattle industry, especially in dairy cattle agriculture. The bovine livestock industry has also been evolved by more advanced protocols and cryoprotectants that has resulted in the improvements of the fertility of frozen-thawed sperm (4–6).

Harsh procedures of cooling, freezing, and thawing cause dramatic changes in the cell which induce injuries to the sperm membrane, thereby reducing sperm quality (7). More specifically, cooling, freezing, and thawing sperm result in high levels of structural and physiological damage from oxidative stress, osmotic injury, and formation of intracellular ice crystals (8–10). All of these contribute to the decrease in fertility, largely due to the impairment of the cell membrane with roughly 50% reduction in viability and motility as well as acrosome integrity (11–13). Optimal freezing temperatures and rates rather than extreme fast and slow seem to be required to avoid the formation of intercellular ice crystals, and to achieve post-thaw survival because more than half of the cryopreserved sperm die post-thaw (14–16). These freezing-induced changes disrupt cell membranes (17) and oxidation of lipids caused by cryopreservation techniques that stimulate the production of reactive oxygen species (ROS) by generating free radicals and osmotic stress resulting in DNA fragmentation in sperm (18–20).

Oxidative stress is a potential factor for poor fertility status with abnormal sperm parameters and indicates the imbalance between the overproduction of ROS and total antioxidant capacity in the cell. Oxidative stress gives rise to impaired sperm function by causing DNA damage, thus remains a challenging factor for male infertility and potential pregnancy losses (21–23). At the physiological concentrations, ROS is necessary for aerobic metabolism, membrane fluidity, and sperm fertilizing ability, while also critical for acrosome reaction and capacitation by modulating cAMP synthesis and tyrosine phosphorylation, and by signaling to support fertilization (24–26). Overproduction of ROS that exceeds the antioxidant capacity of the seminal plasma deteriorates sperm function (27, 28). The procedures of sperm cryopreservation induce production of free radicals caused by the removal of the great majority of seminal plasma, cold shock, and osmotic stress as well as the ubiquitous presence of dead and damaged spermatozoa (29–31).

Despite the significance of sperm freezability affecting bull fertility and its economic impact on cattle farming, there exists no optimal extenders and no conventional methods to accurately evaluate sperm freezability. These gaps in the knowledge and technology bases are important because they are preventing advances both in cryobiology of mammalian male gamete and in the ART. Causes underlying differing freezability remain to be elusive, although physical damage to the membranes and biochemical changes have been associated with cryo-induced oxidative stress (32–34). The overall goal of this study was to test the central hypothesis that sperm cellular and functional phenomes are associated with sperm integrity and freezability.

The specific objective was to ascertain the cellular and functional parameters as well as ROS related changes in sperm from bulls with varied sperm freezability phenotypes. The results of this study advance our understanding of sperm cryopreservation and the development of more integrative methods for precision reproduction through applied biology.

## Results

### Principal Component Analysis for Functional Parameters of Freezability

**Figure 1:**
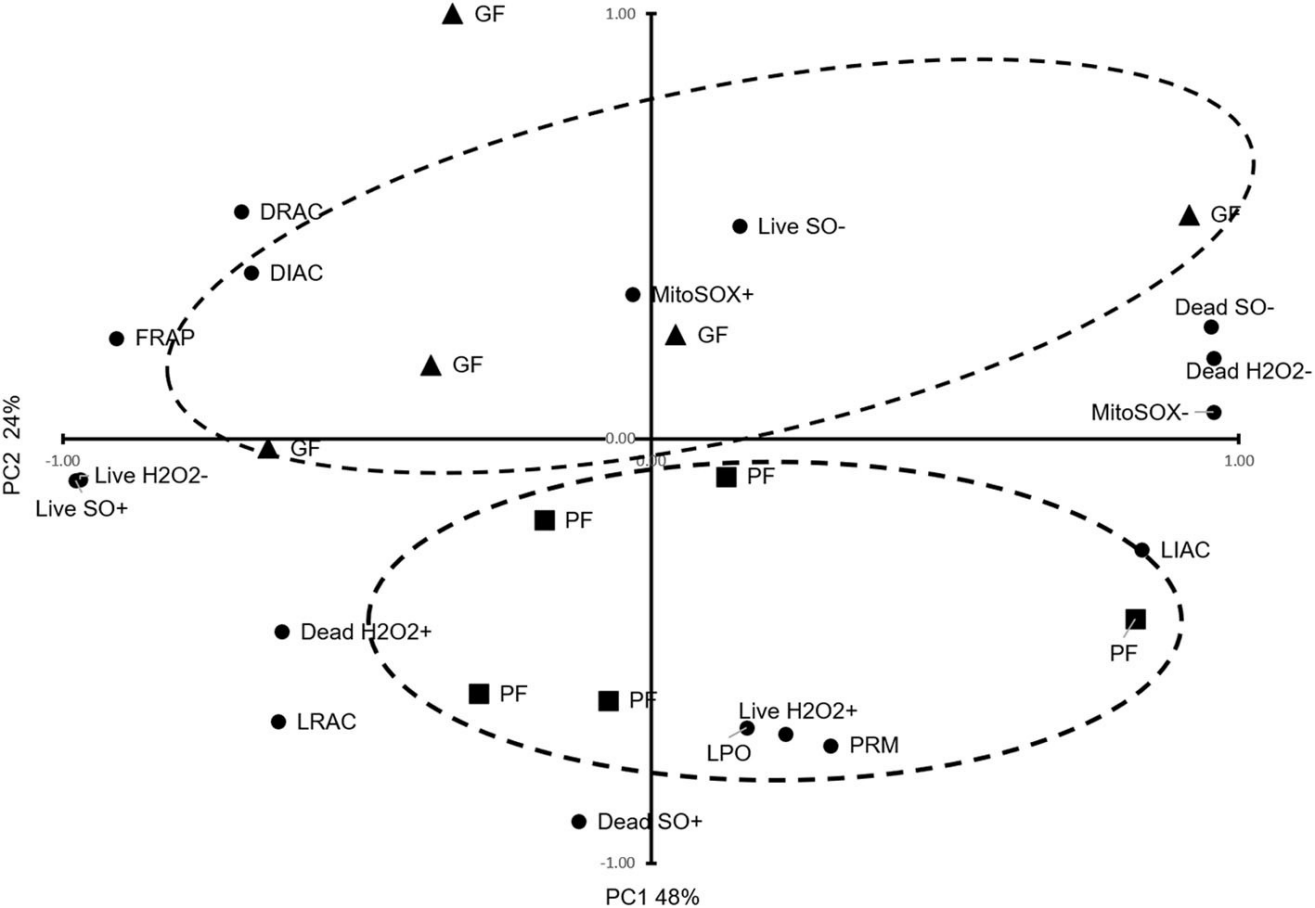
Principal Component Analysis for functional parameters of freezability. The dispersion of bulls regarding important sperm functional parameters, TAC, oxidative parameters, PRM deficiency, and freezability status are provided. The dashed ellipses represent good freezability (GF) and poor freezability (PF).

Using PCA, we reduced the variables to two principal components, PC1 explaining 48% of the variances and PC2 explaining 24% of the variances, and clustered animals into two distinct groups of GF and PF, with an outlier for the GF (Figure 1). Based on the patterns of these variables, PC1 was primarily correlated with FRAP (*r* = 0.91; *P* = 0.0002), live H_2_O_2_− (*r* = 0.97; *P* < 0.0001), dead H_2_O_2_− (*r* = 0.96; *P* < 0.0001), dead H_2_O_2_+ (*r* = 0.63; *P* = 0.052), live SO+ (*r* = 0.98; *P* < 0.0001), dead SO– (*r* = 0.95; *P* < 0.0001), DIAC (*r* = 0.68; *P* = 0.031), and DRAC (*r* = 0.70; *P* = 0.025), LIAC (*r* = −0.84; *P* = 0.003), LRAC (*r* = 0.63; *P* = 0.049), and MitoSOX– (*r* = 0.96; *P* < 0.0001). From the PCA patterns, PC2 was the component that separated the GF and PF groups. It was correlated with LPO (*r* = 0.68; *P* = 0.030), live H_2_O_2_+ (*r* = 0.70; *P* = 0.025), dead SO+ (*r* = 0.90; *P* = 0.0003), and PRM (*r* = 0.72; *P* = 0.0176).

### Sperm Total Antioxidant Capacity (TAC) and Oxidative Parameters in GF and PF Bulls

By assessing the total antioxidant capacity (TAC) in the GF and PF bulls, the current study revealed that the FRAP values of GF and PF were similar (55.26 and 63.35 μM, respectively; *P* = 0.462). Lipid peroxidation (LPO), as a second messenger of oxidative stress, in bull sperm was measured by the reaction of thiobarbituric acid (TBA). The difference between the GF and PF bulls was similar for lipid peroxidation (4.57 and 3.98 μM, respectively; *P* = 0.272). The percentages of live hydrogen peroxide negative (Live H_2_O_2_−) and live hydrogen peroxide positive (Live H_2_O_2_+) sperm cells did not differ between the GF and PF bulls, respectively (47.65 and 47.96%, *P* = 0.974) and (1.10 and 0.012%, *P* = 0.364). Likewise, percentages of dead H_2_O_2_ negative (Dead H_2_O_2_−) and dead H_2_O_2_ positive (Dead H_2_O_2_+) sperm were similar GF and PF bulls, respectively (51.02 and 51.98%, *P* = 0.92) and (0.19 and 0.03%, *P* = 0.16). As for the assessment of superoxide anion (SO) in semen from GF and PF bulls, the percentages of live superoxide anion (Live SO–) and live superoxide anion positive (Live SO+) were similar between the freezability groups, respectively (0.03 and 0.28%, *P* = 0.284), (48.44 and 49.84%, *P* = 0.878). The percentage of dead SO– (Dead SO–) were similar between the GF and PF groups (35.79 and 37.93%, respectively; *P* = 0.823), while the dead superoxide anion positive (Dead SO+) sperm in the GF bulls were greater than in the PF (15.72 and 12.00%, respectively; *P* = 0.024). The levels of mitochondria-specific reactive oxygen species were measured in sperm from bulls with different sperm freezability phenotypes. The percentages of mitochondrial negative ROS (MitoSOX–) and positive ROS (MitoSOX+) were similar between the GF and PF, respectively (47.33 and 43.13%, *P* = 0.686) and (1.79 and 2.59%, *P* = 0.489) as depicted in Table 1.

**Table 1:** Sperm total antioxidant capacity (TAC), lipid peroxidation (LPO)-oxidative parameters in good and poor freezability bulls.

**TAC and oxidative parameters**	**Good freezability** **(*n* = 5)**	**Poor freezability** **(*n* = 5)**	**Significance**
LPO (μM)	4.57 ± 0.29	3.98 ± 0.41	*P* = 0.272
FRAP (μM)	55.26 ± 8.16	63.35 ± 6.58	*P* = 0.462
Live H_2_O_2_− (%)	47.65 ± 7.12	47.96 ± 5.92	*P* = 0.974
Live H_2_O_2_+ (%)	1.10 ± 1.07	0.012	*P* = 0.364
Dead H_2_O_2_− (%)	51.02 ± 7	51.98 ± 5.94	*P* = 0.92
Dead H_2_O_2_+ (%)	0.19 ± 0.10	0.03 ± 0.01	*P* = 0.16
Live SO– (%)	0.03 ± 0.02	0.28 ± 0.20	*P* = 0.284
Live SO+ (%)	48.44 ± 7.12	49.84 ± 5.23	*P* = 0.878
Dead SO– (%)	35.79 ± 7.76	37.93 ± 5.10	*P* = 0.823
Dead SO+ (%)	15.72 ± 1.09	12.00 ± 0.78	*P* = 0.024
MitoSOX– (%)	47.33 ± 9.21	43.13 ± 4.04	*P* = 0.686
MitoSOX+ (%)	1.79 ± 0.67	2.59 ± 0.87	*P* = 0.489

*The data are presented as mean ± SEM. FRAP, ferric reducing antioxidant power*.

### Sperm Functional Parameters in GF and PF Bulls

Analyses of data on sperm functional parameters revealed that GF had greater PTV than PF (63.83 and 52.10%, respectively; *P* = 0.0001). The results generated by the pattern of forward and side scatter (FSC-SSC) light revealed the acrosome status of sperm from different freezability bulls. Sperm samples from PF bulls had greater dead intact acrosome (DIAC) than those from the GF bulls (26.29 and 16.10%, respectively; *P* = 0.028) whereas GF bulls had almost greater live intact acrosome (LIAC) than the PF bulls (64.47 and 50.05, respectively; *P* = 0.084). There was also greater dead reacted acrosome (DRAC) in PF bulls compared to those from the GF (19.27 and 11.48%, respectively; *P* = 0.007). However, live reacted acrosome (LRAC) percentages were similar between the GF and PF bulls (7.54 and 4.10%, respectively; *P* = 0.17). We also demonstrated through protamine deficiency assay that the levels of PRM were similar between the GF and PF bulls (7.20 and 0.64%, respectively; *P* = 0.206) as depicted in Table 2.

**Table 2:** Sperm functional parameters in the GF and PF bulls.

**Sperm functional parameters**	**Good freezability (*n* = 5)**	**Poor freezability (*n* = 5)**	**Significance**
PTV (%)	63.83 ± 0.76	52.10 ± 1.29	*P* = 0.0001
DIAC (%)	16.108 ± 1.47	26.29 ± 3.50	*P* = 0.028
DRAC (%)	11.48 ± 1.78	19.27 ± 1.27	*P* = 0.007
LIAC (%)	64.47 ± 4.80	50.05 ± 5.50	*P* = 0.084
LRAC (%)	7.54 ± 1.85	4.10 ± 1.33	*P* = 0.17
PRM (%)	7.20 ± 4.77	0.64 ± 0.06	*P* = 0.206

*Sperm functional parameters are presented for good freezability (n = 5) and poor freezability (n = 5) bulls. Data are expressed as mean ± SEM. DIAC, dead intact acrosome; DRAC, dead reacted acrosome; LIAC, live intact acrosome; LRAC, live reacted acrosome reacted; PRM, protamine deficiency*.

### Associations Between Post-thaw Viability and Sperm Characteristics

**Figure 2:**
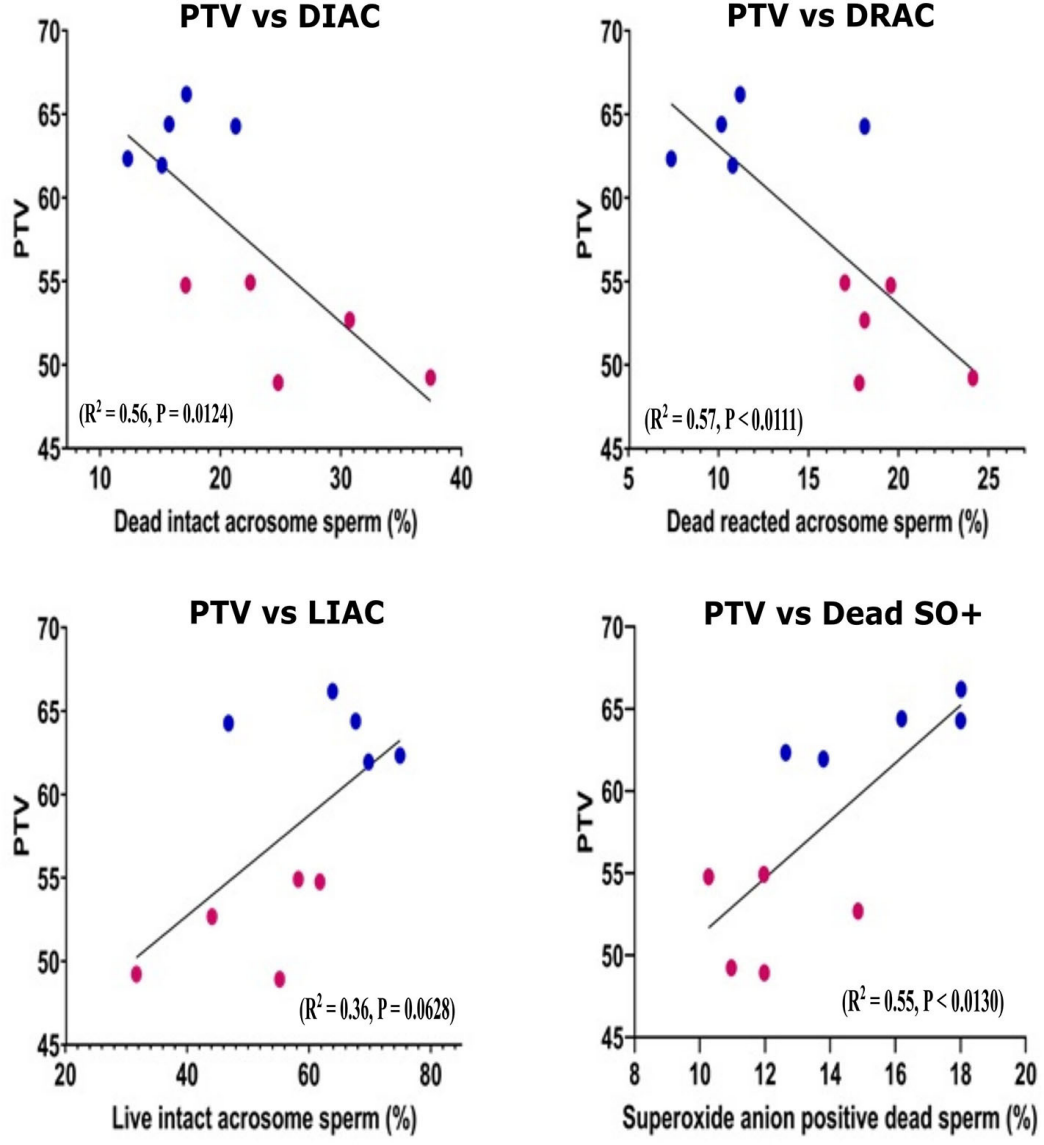
Scatter plot distribution of different sperm parameters. Scatter plot distribution of different sperm parameter in good (*n* = 5) and poor (*n* = 5) freezability bulls. Lower (claret) dots: PF bulls, upper (blue) dots: GF bulls. The Pearson correlation is indicated. PTV, Post thaw viability; DIAC, dead intact acrosome; DRAC, dead reacted acrosome; LIAC, live intact acrosome; LRAC, live reacted acrosome; Dead SO+, dead superoxide anion.

Pearson correlation was performed for the sperm parameters between the GF and PF groups. Using scatter plots, we demonstrated the distributions of bulls for sperm functional parameters in relation to freezability status. The number of cells with dead intact acrosome (DIAC) (*R*^2^ = 0.56, *P* = 0.0124) and dead reacted acrosome (DRAC) (*R*^2^ = 0.57, *P* < 0.0111) were negatively correlated with freezability phenotype. The number of cells with live intact acrosome (LIAC) (*R*^2^ = 0.36, *P* = 0.0628) and Dead SO+ (*R*^2^ = 0.55, *P* < 0.0130) were positively correlated with freezability (Figure 2). The nonparametric measure of spearman rank correlation of PTV with live H_2_O_2_+, dead H_2_O_2_+, live SO–, and PRM in GF (*n* = 5) and PF (*n* = 5) were performed for freezability bulls. The PTV was significantly correlated with live H_2_O_2_+ (r_s_ = 0.63, *P* = 0.0498), and PRM (r_s_ = 0.70, *P* = 0.0251) whereas dead H_2_O_2_+ (r_s_ = 0.26061, *P* = 0.4671) and live SO– (r_s_ = −0.28049, *P* = 0.4325) have no significant association with PTV.

## Discussion

The cellular and functional health of sperm in cryopreservation, as well as dynamics of ROS and antioxidant capacity, are poorly defined. Lack of such knowledge is an important problem because it hinders the understanding process in cryobiology of mammalian male gametes and the advancement of ART. The overarching goal of this study was to ascertain cellular and functional phenomes associated with sperm integrity and freezability using methods in contemporary biology and sperm from phenotypically distinct bulls.

In this study, the two principal components explained 72% of the variance of PTV which clustered animals into two distinct groups of GF and PF, with an outlier for the GF. PC1 explaining 48% of variance correlated with FRAP, live H_2_O_2_−, dead H_2_O_2_−, dead H_2_O_2_+, live SO+, dead SO–, MitoSOX–, DIAC, DRAC, and LIAC. This showed that total antioxidant capacity and acrosome status as well as some oxidative parameters define PC1. However, PC2 explained 24% of the variance, which was correlated with LPO, live H_2_O_2_+, dead SO+, and LRAC, and PRM. These findings showed that lipid peroxidation, oxidative parameters, and protamine deficiency status accounted for the variance in PC2 (Figure 1). The PCA suggests that a combination of analyses from different variables of cellular and functional parameters as well as oxidative parameters can help distinguish the freezability status of bulls.

Sperm are susceptible to oxidative stress due to the presence of large amounts of polyunsaturated fatty acids in sperm membranes as potential targets of oxidative stress (35). In addition, the sperm cytoplasm is small and contains low levels of protective enzymes and antioxidants. As such, sperm membranes are prone to LPO during freezing and thawing cycles (27). In our study, we did not find a correlation between LPO and freezability phenotypes, and the concentration was similar between GF and PF bulls (Table 1). In support of these findings, LPO was not related to freezing-thawing of fresh and frozen human sperm (36). In rams, LPO did not change during cooling and thawing procedures of cryopreservation (37). Moreover, young and old buffalo bulls had similar levels of lipid peroxidation in fresh vs. frozen-thawed sperm (38). It was shown that lipid peroxidation of bull sperm has no relationship with the concentration of some of the enzymatic antioxidants (39). However, Gürler et al. (40) reported that SOD and LPO were positively correlated, which was not anticipated because SOD causes dismutation of O_2_ which then renders to H_2_O_2_. They also, in similar lines with literature, found a negative relationship between LPO and total antioxidant capacity 3 h after thawing and stimulation of LPO by t-butyl hydroperoxide. We inferred that sperm cells still maintain the balance between TAC and oxidative stress induced LPO for a while and without any induction. Therefore, our findings in LPO experiments may have resulted from the stability of the sperm membrane counteracting the lipid compositions during freezing-thawing.

The antioxidant systems in sperm are of cytoplasmic origin and counterbalance the detrimental effects of cryopreservation which results from lipid peroxidation and ROS (41, 42). In our study, there was no correlation between TAC and freezability phenotypes, and the concentrations were similar between GF and PF bulls (Table 1). Although the parameters of enzymatic antioxidant defense mechanisms were not evaluated in our study, the enzymatic antioxidant defenses play important roles against lipid peroxidation and that the seminal plasma is the main source of antioxidants (43, 44). However, it was reported that GPX and SOD enzymes are not sufficient to alleviate cryodamage because intercellular antioxidants may support only physiological changes in sperm cells (32).

Production of ROS is one of the primary underlying reasons for sperm damage in cryopreservation which in turn impairs post-thaw motility, viability, and other sperm parameters (45, 46). However, the exact involvement of H_2_O_2_ and superoxide anion (SO) in male fertility remains elusive. In bovine sperm cells, Gürler et al. (33) showed that H_2_O_2_ causes DNA damage without any changes in other ROS and sperm viability. Unexpectedly, while the levels of live H_2_O_2_+ were similar between the freezability groups (Table 1), PTV was correlated with live H_2_O_2_+, implying that a higher percentage of viable sperm cells in the GF bulls might still be producing ROS without compromising sperm freezability.

The ATP is produced by oxidative phosphorylation catalyzed by the respiratory chain in the mitochondria which generate ROS, adequate levels of which are required for sperm hyperactivation, capacitation, acrosome reaction, and for the fusion of spermatozoon and oocyte (47, 48). On the other hand, excessive production of ROS can be detrimental for proteins and membrane lipids (49). In our study, we found that mitochondrial ROS generation was not associated with PTV status in sperm from the bulls with different sperm freezability phenotypes. Because there was no significant difference among both groups in mitochondrial oxidative stress (Table 1), we inferred the machinery was able to maintain a balance between generation and removal of ROS by mitochondrial enzymatic antioxidant in the mitochondrial matrix. This may lead to a small amount of H_2_O_2_ released into the extracellular (50) which was consistent with our result. This was supported by Armstrong et al. (51) and De Lamirande and Gagnon (52) that action of H_2_O_2_ in human sperm is not dependent on mitochondrial oxidative phosphorylation. In our study, the probe used reacts with superoxide in viable cells of mitochondria which actively respires. Our study may explain that optimal intercellular ROS production by mitochondria could be maintained while sperm confront cryopreservation. Due to the fact mitochondrial ROS is produced through metabolic substrates and the extracellular environment including mitochondrial membrane potential (53, 54), rather than through cryodamage. Disruption of mitochondria is likely to lead to an increased production of cellular ROS in sperm causing an increase in superoxide anions in frozen-thawed sperm as compared to fresh sperm (50, 55). In our study, live negative and positive, and dead superoxide anion negative sperm cells were not correlated with PTV, while dead superoxide anion positive (SO+) cells correlated with PTV (Figure 2) as well as with higher levels in GF than PF bulls (Table 1). Interestingly, it was demonstrated that increased concentrations of sperm contribute to higher superoxide anion generation (56). Likewise, in fresh sperm, a reduction in sperm concentration was found to alleviate oxidative stress in bulls (57). This is likely because freezability may also be affected by some other factors of cryopreservation, such as seasonal variations, cooling–thawing rates, sperm source, and type of extender or cryoprotectants.

While cryopreservation induces production of ROS by increasing superoxide anion, acrosome reaction is linked to superoxide anion production. The acrosome reaction is correlated with the generation of extracellular O_2_− and H_2_O_2_ and that low levels of ROS function as signaling transducers and are critical for the initiation of acrosome reaction (58, 59). On the other hand, excessive ROS harms the acrosome membrane, as an example, sperm acrosome integrity injuries occurred after cryopreservation that led to cryocapacitation thus premature acrosome reaction due to abnormalities in membrane fluidity (60, 61). Our findings that dead intact (DIAC) and dead reacted acrosome (DRAC) were negatively correlated with the freezabilitiy phenotype of PTV (Figure 2). Additionally, PF bulls had higher DIAC and DRAC than GF bulls (Table 2). Our study showed that proportions of live intact acrosome were closely related to freezability, however, levels of LRAC were similar between the GF and PF bulls (Table 2). This is consistent with reports that intact acrosome is an important factor for the quality of frozen sperm cells in bulls (62) as well as for the success of fertilizing capacity of cryopreserved bovine sperm (63). However, live reacted acrosome was not associated with PTV and was similar between the GF and PF bulls (Table 2). Acrosome reaction was claimed to be better suitable for evaluating frozen-thawed sperm before keeping males as breeding bulls (64) and in ram, acrosomal integrity was linked with PTV, therefore, results of our study elucidate acrosomal integrity as an integral part of freezability, and support findings that the physiological ROS are essential for acrosome reaction.

Impaired and deficient protamination is an indication of chromatin immaturity that can influence sperm function (65–67). Therefore, we employed CMA3 that competes with protamines for binding to the minor groove of DNA which indirectly shows the levels of protamine deficiency in the sperm chromatin. Sperm protamine content measured in human and bull indicates that sperm protamine deficiency can be linked to DNA damage (68–70). In addition, freeze-thaw may impair the bonding of disulfide bridges in protamine, thus lead to increased DNA damage (71), protamine deficiency did not always result in DNA damage though (72). On the other hand, it is also intriguing that protamine deficiency was found to be low in bull compared to human (73). This seems to be the likely reason that bull sperm are more resilient against the detriments of freezing-thawing protocol (74). In contrast, results of our study revealed that the percentage of protamine deficiency did not change in GF and PF sperm, but correlated with freezability status (Table 2) which is supported by the previous reports that protamine deficiency did not differ between fresh and freeze-thawed spermatozoa (75). Variations in protamine contents are detectable in sperm from different bulls sperm (76) and that sperm from different aged bulls had distinct protamine deficiency levels (74).

A comprehensive evaluation of the sperm cellular and functional physiopathology is fundamental to the male reproduction research to advance knowledgebase in cryobiology through elucidating and improving the freezability status. In addition to the physiological role of ROS, the structural integrity of sperm organelles such as the acrosome is more important for assessing proper sperm functions. The limitations of this study were that because of the lack of feasibility factors, we were unable to analyze the fresh samples before freezing, and that the sample size could be increased. However, through integrative approaches and analyses of cells with phenotypes, we demonstrated the cellular and functional dynamics of sperm stemming from cryodamage that can affect sperm physiology and reproductive health.

## Materials and Methods

### Semen Collection and Determination of Bull Semen Freezability

**Figure 3:**
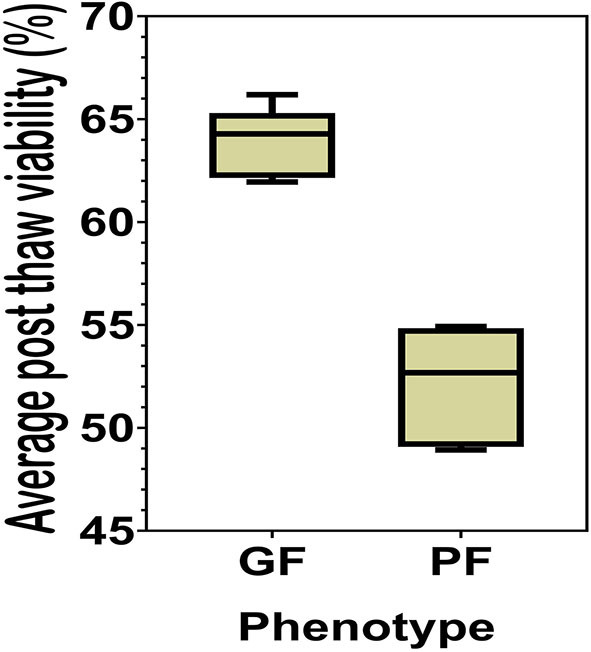
Staining of sperm cells with SYBR-14 and PI indicating viability. Representative image of sperm stained with SYBR-14 and PI indicating viability viewed with fluorescence microscope (20×) is provided. Arrows indicate the viable cells stained green with SYBR-14; asterisks indicate dead cells stained red with PI; and the moribund sperm stained with both colors.

**Figure 4:**
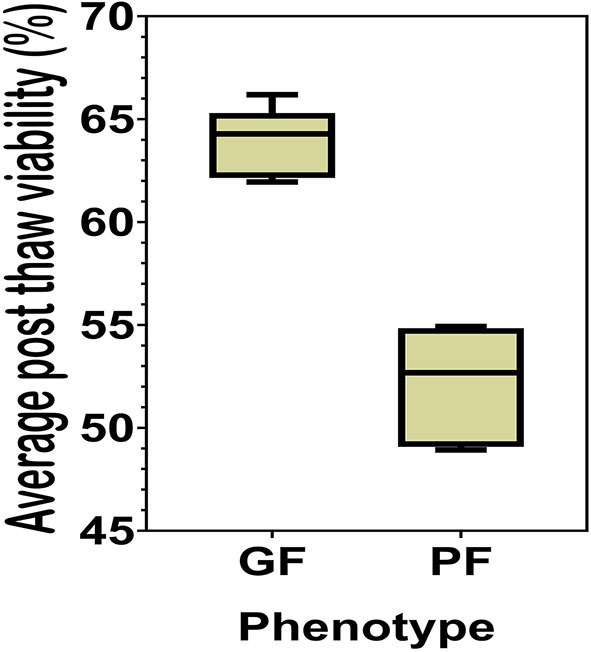
Percent averages of GF and PF phenotype bulls. The average post-thaw viability in bulls from the categories of the Good freezability phenotype and the Poor freezability phenotype. Bulls were arbitrarily selected and placed into one of the two categories based on the percent average of post-thaw viability from the population average (*P* < 0.0001).

The sperm samples analyzed in this study were obtained from Alta Genetics Inc. (Watertown, WI); therefore, the experiments conducted in our laboratories did not involve live animals. Five bulls were assigned to a good freezability group (GF); whereas the other five were assigned to poor freezability group (PF) according to the post-thaw viability (PTV) data provided by Alta Genetics Inc. Post-thaw viability of sperm was assessed using a fluorescent stain combination of SYBR-14 with propidium iodide (SYBR-14/PI, Live/Dead Sperm Viability Kit L-7011, Thermo Fisher Scientific) as described previously (Figure 3) (16, 77). Collectively, a unique freezability phenotype was generated to characterize variations among bulls for their lifetime PTV of sperm. For this research, we used post-thaw viability data generated over 8 years between 2008 and 2016. The database included 100,448 ejaculates from 860 Holstein bulls that were collected at least 20 different times in ~3 months. The average and standard deviations of PTV for the individual bull were calculated, and bulls were ranked based on average PTV, which represents the freezability score. The average post-thaw viabilities of all bulls of 10 bulls ranged from 48.93 (*n* = 5) to 66.19% (*n* = 5) (population average 57.9 ± 6.5%) (*P* = 0.0001) (Figure 4). The bulls were then arbitrarily classified as GF and PF based on average post-thaw viability score and the difference from the population average (Table 3). The bulls were housed in the same nutrition and management environments to prevent sample variations. Semen from 10 bulls (GF, *n* = 5; PF, *n* = 5) was collected using an artificial vagina and processed immediately. Bull semen was preserved in a commercial Egg-Yolk-Tris based extender and frozen at Alta Genetics' laboratories using standard protocols (78). Cryopreserved sperm samples were shipped to Mississippi State University (MSU) in a liquid nitrogen containing tank.

**Table 3:** Bull freezability phenotype information.

**Bull ID**	**Freezability status**	**Average post-thaw viability (%)**	**% Post-thaw viability difference from population average**
1	Good freezability	66.19	8.23
2		64.4	6.44
3		64.28	6.32
4		62.34	4.38
5		61.95	3.99
6	Poor freezability	54.92	−3.04
7		54.77	−3.19
8		52.68	−5.28
9		49.23	−8.73
10		48.93	−9.03

*Bulls 1–5 were defined as high freezability (HF) and bulls 6–10 were grouped as poor freezability (PF). The average post-thaw viability scores and the differences from the population average are presented*.

### Thiobarbituric Acid Reactive Substances (TBARS) Assay

For thiobarbituric acid reactive substances (TBARS) assay, sperm were thawed at 37°C for 30 s and then the extender was removed by centrifugation at 800 × *g* for 10 min. Sperm were washed twice with PBS and diluted to a concentration of 5 × 10^6^ cells in 100 μL of PBS solution. Thiobarbituric acid reactive substances assay was employed to evaluate lipid peroxidation by measuring malondialdehyde (MDA) produced from the oxidation of polyunsaturated fatty acids in the sperm according to the previously described method (79). Briefly, 100 μL of sperm were mixed with 300 μL stock solution (TCA-TBA-HCl, 15% trichloroacetic acid, 0.375% thiobarbituric acid, and 0.25 N HCl) and the mixture was heated in 90°C water bath for 30 min, with gentle rocking once every 10 min, followed by quick cooling on ice-water for 5 min. The mixture was then centrifuged at 10,000 × *g* in 4°C for 10 min to pellet the precipitated proteins. The clear supernatant (200 μL) was collected and transferred into a 96-well plate (Santacruz, CA, USA, #sc-204463), together with an MDA calibration curve. The absorbance was measured at 532 nm in a microplate reader (FlexStation® 3 Benchtop Multi-Mode Microplate Reader, Orleans Drive, Sunnyvale, CA, USA). The TBARS value was expressed as μM of MDA.

### Ferric-Reducing Antioxidant Power Assay (FRAP)

Cryopreserved sperm in straws were thawed the same way as above and centrifuged at 500 × *g* for 6 min to remove the extender. The pellet was washed twice with PBS and suspended in modified Tyrode Hepes-buffered medium (sp-TALPH) at a concentration of 5 × 10^6^ cells in 200 μL. Antioxidants in sperm were extracted by sonication (Fisher Scientific, CPX962217R, PA, USA). The total antioxidant capacity (TAC) was assessed by the method of the ferric reducing antioxidant power (FRAP) (80). The FRAP solution [300 mM acetate buffer (pH 3.6), 10 mM 2,4,6-tripyridyl-S-triazine (TPTZ) in 40 mM HCl, and 20 mM FeCl_3_.6H_2_O in the ratio of 10:1:1] was mixed with 200 μL sperm cell extract and vortexed. The absorbance was measured at 593 nm against a reagent blank and absorbance of a standard solution (FeSO_4_.7H_2_O). The FRAP value was expressed as μM of FeSO_4_.7H_2_O.

### Assessment of Reactive Oxygen Species (ROS)

**Figure 5:**
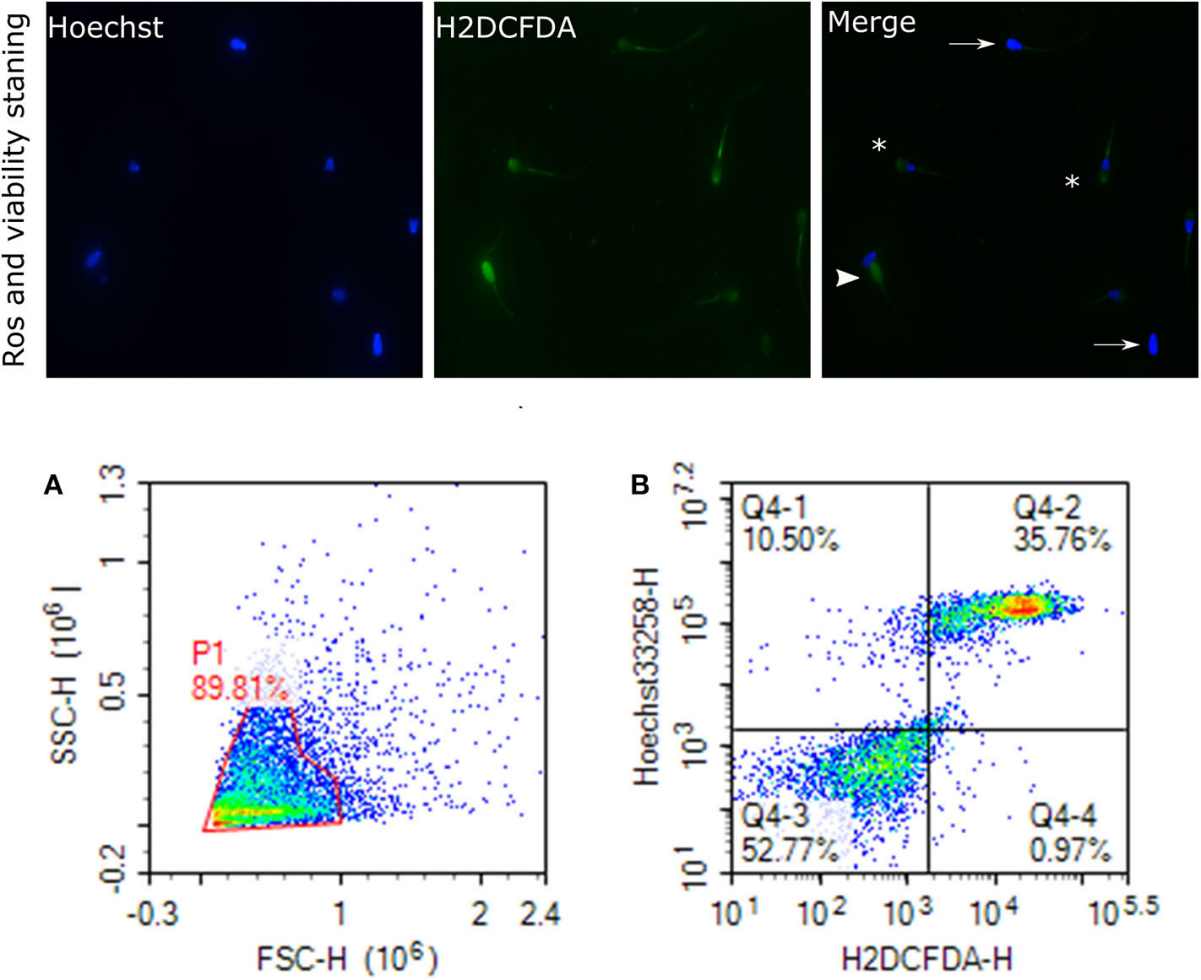
Reactive oxygen species (H_2_O_2_) and viability. Representative images of sperm stained with H_2_DCFDA and Hoechst indicating ROS and live cells, viewed with fluorescence microscope (60×) are depicted. Arrows indicate the live cells stained with uniform blue Hoechst; asterisks indicate the ROS stained and live cells; arrowhead show the dead and ROS stained cells. This figure is presented as two categories because two ROS probes were used. **(A)** gated sperm cell population. **(B)** Q1 viable cell H_2_O_2_ negative cells, Q2 viable H_2_O_2_ positive, Q3 nonviable H_2_O_2_ negative, and Q4 nonviable H_2_O_2_ positive.

Sperm pellet was washed twice in 1 mL of PBS at 800 × *g* for 10 min. Also, 2 × 10^6^ sperm cells were suspended in 300 μL of PBS for the flow cytometry assays, including mitochondrial ROS, acrosome reaction, and protamine deficiency assay. Sperm ROS were measured by following a previously reported protocol (81), with some modifications. Briefly, 10 μL of 2 mM 2′,7′-dichlorodihydrofluorescein diacetate (H_2_DCFDA, Invitrogen, D399), 10 μL of 40 μM dihydroethidium (DHE; Invitrogen, D11347), and 10 μL of 40 μM Hoechst 33258 (Invitrogen, H3569) were added to the resuspended sperm cells (2 × 10^6^ sperm/mL) and incubated at 37°C in the water bath in the dark for 25 min. Following the incubation, the excitation of Hoechst 33258 was performed with a laser-excited fluorescence channel (VL1) at 405 nm and detected using a 445/45 nm pass filter. The excitation of DHE was performed with laser-excited fluorescence channel (YL2) at 561 nm and detected using a 615 nm pass filter whereas excitation of H_2_DCFDA was performed with laser-excited fluorescence channel (BL1) at 488 nm and detected using a 530 nm filter. A total of 10,000 events was evaluated for each bull sample and cell populations were gated as percentages of live-dead H_2_O_2_+, and live-dead superoxide anion positive and negative (SO+, SO–). Sperm stained with H_2_DCFDA and Hoechst indicating ROS and live cells are depicted in Figure 5. Flow cytometry assays were performed using ACEA NovoCyte® 3000 flow cytometer and NovoExpress software (ACEA) was used for data acquisition and analyses.

### Assessment of Mitochondrial ROS

**Figure 6:**
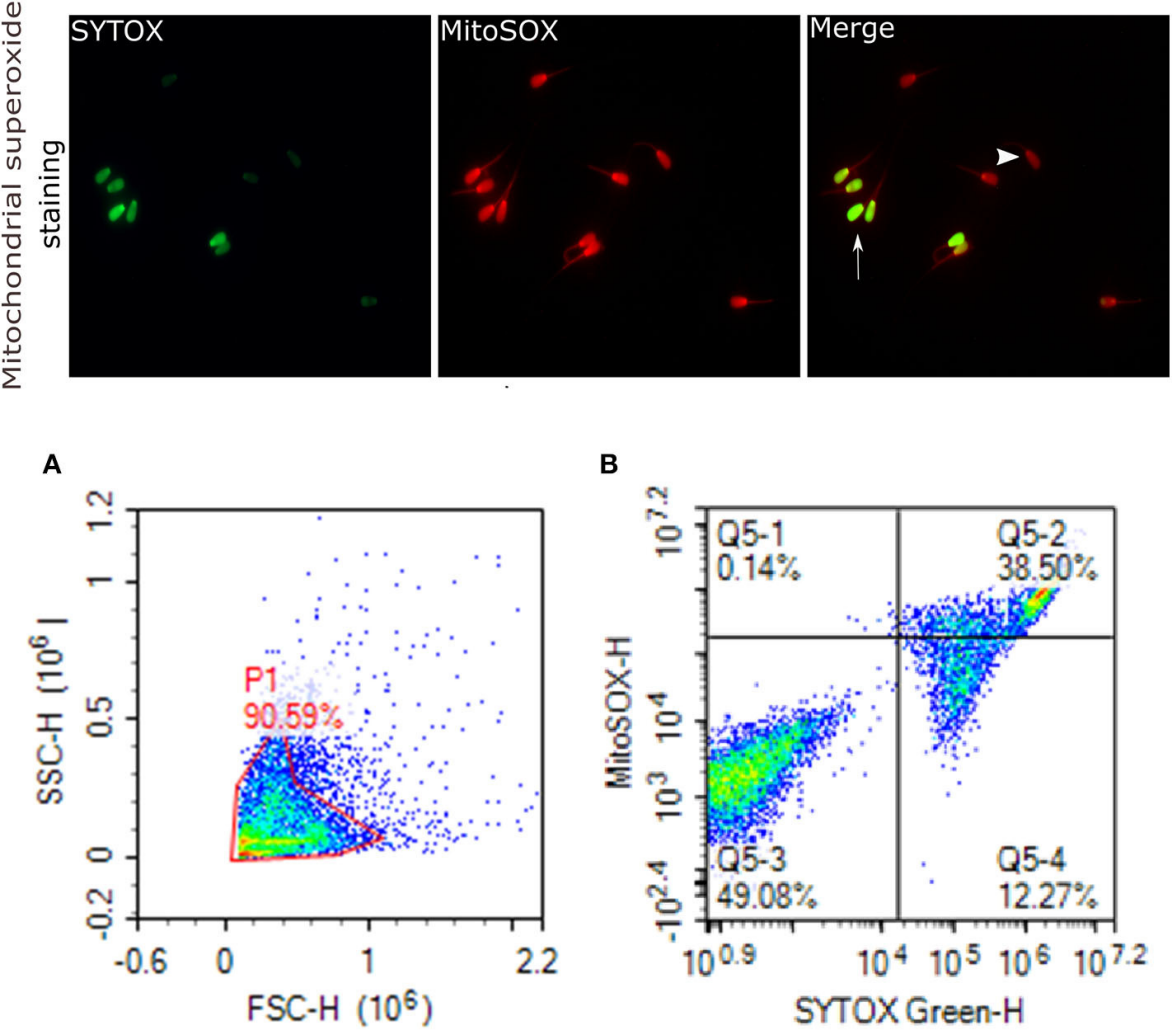
Mitochondrial superoxide generation. Representative images that were viewed with fluorescence microscope (60×) are provided for sperm stained with MitoSOX red for sperm positive mitochondrial superoxide generation, and SYTOX (green) for dead cells. Arrows indicate dead superoxide positive sperm; arrowhead shows live superoxide positive. **(A)** gated sperm cell population. **(B)** Q1 viable MitoSOX+, Q2 dead MitoSOX+, Q3 viable MitoSOX–, Q4 dead MitoSOX–.

Mitochondrial ROS (mROS) levels in sperm cells were determined using a modified method of the fluorescent probe of Mito-SOX™ Red (Molecular Probes, Invitrogen, cat. no. M36008) (82). Briefly, cryopreserved sperm in straws were thawed at 37°C for 30 s and, a total of 2 × 10^6^ cells was used for each analysis. Sperm were incubated in 3 mM of MitoSOX™ Red and 0.05 μM of SYTOX® Green (Molecular Probes, Invitrogen, cat. no. S34860) at 37°C in the dark for 15 min. Following the incubation, samples were then washed using PBS and analyzed using flow cytometer; the excitation of Mito-SOX™ Red was performed with laser-excited fluorescence channel (YL2) at 561 nm and detected using a 620 nm pass filter whereas excitation of SYTOX was performed with laser-excited fluorescence channel (BL1) at 488 nm and detected using a 530 nm filter. A total of 10,000 events was counted after gating as percentages of live MitoSOX– and MitoSOX+. Sperm stained with MitoSOX red for mitochondrial superoxide generation, and SYTOX (green) for dead cells are depicted in Figure 6.

### Acrosome Integrity Assay

**Figure 7:**
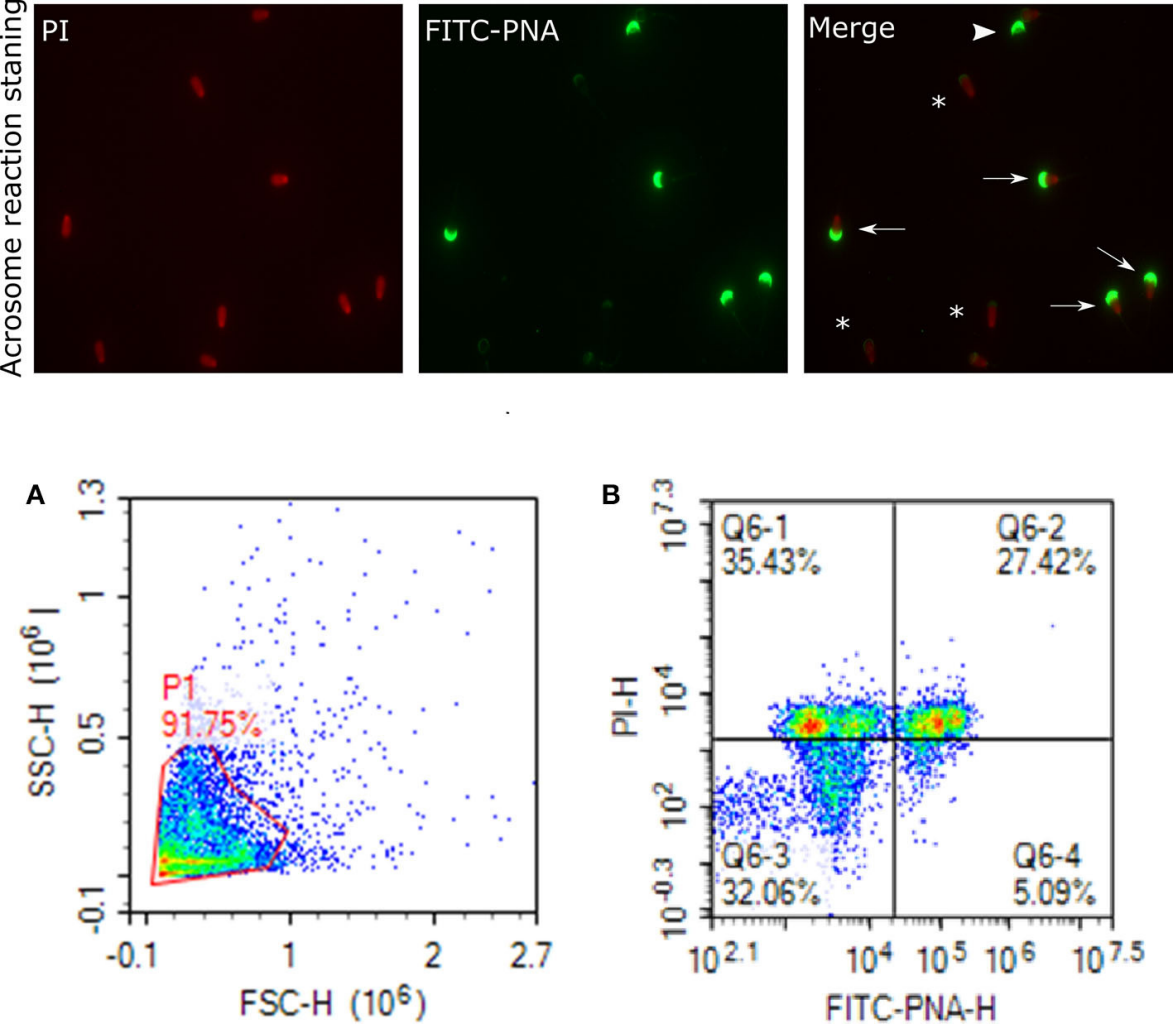
Acrosome staining of sperm cells. Representative images of sperm stained with FITC-PNA and PI indicating acrosome and dead cells, viewed with fluorescence microscope (60×). Arrows indicate dead and intact acrosome cells with uniform green FITC-PNA fluorescence of acrosome site; asteriks indicate acrosome-reacted cells with fluorescence along its outline; arrowhead shows viable cells stained with uniform green FITC-PNA fluorescence of acrosome. **(A)** gated sperm cell population. **(B)** Q1 dead intact acrosome (DIAC), Q2 dead reacted acrosome (DRAC), Q3 live intact acrosome (LIAC), live reacted acrosome (LRAC).

Status of the acrosome reaction was evaluated using fluorescein isothiocyanate-peanut agglutinin (FITC-PNA; Sigma- Aldrich, St. Louis, MO) according to the methods described by (83), with modifications. Briefly, 10 μL of FITC-PNA and 1.2 μL of PI (2.4 mM) were mixed with 300 μL of 2 × 10^6^ cells and then incubated at 37°C in a water bath in the dark for 20 min. The excitation of FITC-PNA was performed with laser-excited fluorescence channel (BL1) at 488 nm and detected using a 530 nm filter, and PI was excited at (BL3) at 488 nm and detected using a 620 nm filter. A total of 10,000 events using a flow cytometer was evaluated for each sample. The cell populations were distinguished as live reacted acrosome (LRAC), live intact acrosome (LIAC), dead reacted acrosome (DRAC), and dead intact acrosome (DIAC). Sperm stained with FITC-PNA and PI indicating acrosome and dead cells were viewed using fluorescence microscopy (Figure 7).

### Protamine Deficiency (PRM) Assay

Protamine deficiency assay was performed to assess the extent of replacement of histones by protamines using chromomycin A3 (CMA3) according to a previously reported assay (70). Sperm pellets containing 2 × 10^6^ sperm cells were resuspended in TNE buffer (10 mM Tris, 1 mM EDTA, 100 mM NaCl) after thawing and centrifugation twice in PBS at 5,000 × *g* at room temperature for 15 min. The CMA3 solution (0.25 mg/mL) was prepared by dissolving CMA3 (Sigma-Aldrich) in McIlvaine's buffer (LabChem, Inc. LC163004); 200 μL of which were added to spermatozoa. The resuspended sperm was incubated at 37°C in a water bath in the dark for 20 min and washed twice in PBS by centrifugation (500 × g at room temperature for 10 min). Sperm cells were gated using FSC and SSC and identified by PI-positive signal excited using the fluorescence channel (BL3) at 488 nm. The signals were detected using a 620 nm filter whereas CMA3 was excited using a laser-excited fluorescence channel (VL2) at 405 nm and detected with a 530 nm pass filter. For each bull sample, a minimum 10,000 of events was assessed. The percentage of CMA3 positivity was expressed as median fluorescence intensity (MFI) read from the histogram, and measured by the ratio between the MFI of each sample and MFI of negative cells.

### Statistical Methods

Principal component analysis (PCA) of standardized data (mean-centered and divided by the standard deviation of each variable) was used to reduce sperm functional parameters and oxidative stress parameters to two principal components (PC) while maintaining total variance in data. The loadings, representing correlation coefficients of variables with principal component 1 (PC1; horizontal coordinate) and principal component 2 (PC2; vertical coordinate), were used to map these variables in a biplot. The PC scores or rankings were used to map bulls having different freezability phenotype on the same biplot. The CORR procedure of SAS for Windows v9.4 (SAS Institute, Inc., Cary, NC) was used to determine Pearson and Spearman's correlation coefficients (in case of non-parametric data) between post-thaw viability with sperm functional and oxidative stress parameters. The *t*-test procedure of SAS 9.4 was used to conduct a two-sample *t*-test. For non-normally distributed outcomes, Wilcoxon rank-sum tests were performed using Proc NPAR1WAY. The actual probability values of statistical significance were reported.

## Data Availability Statement

The raw data supporting the conclusions of this article will be made available by the authors, without undue reservation.

## Author Contributions

MH, TD, EM, and AK: conception and design of the study. AK, ET, MH, DS, and TD: sample acquisition and processing. MH, MU, TD, DS, EM, AK, and WT: acquisition, analysis, and interpretation of data. MH, MU, TD, AK, and EM: drafting the article or revising it critically for important intellectual content. All authors contributed to the article and approved the submitted version.

## Conflict of Interest

ET employed by the company URUS Group LP. The remaining authors declare that the research was conducted in the absence of any commercial or financial relationships that could be construed as a potential conflict of interest.
